# Subwavelength polarization optics via individual and coupled helical traveling-wave nanoantennas

**DOI:** 10.1038/s41377-019-0186-2

**Published:** 2019-08-28

**Authors:** Mengjia Wang, Roland Salut, Huihui Lu, Miguel-Angel Suarez, Nicolas Martin, Thierry Grosjean

**Affiliations:** 1 FEMTO-ST Institute UMR 6174, Univ. Bourgogne Franche-Comté CNRS, Besancon, France; 2 Guangdong Provincial Key Laboratory of Optical Fiber Sensing and Communications, Department of Optoelectronic Engineering, Jinan University, Guangzhou, 510632 China

**Keywords:** Nanophotonics and plasmonics, Sub-wavelength optics

## Abstract

Light polarization control is a key factor in modern photonics. Recent advances in surface plasmon manipulation have introduced the prospect of more compact and more efficient devices for this purpose. However, the current plasmonic-based polarization optics remain much larger than the wavelength of light, which limits the design degrees of freedom. Here, we present a plasmonic traveling-wave nanoantenna using a gold-coated helical carbon nanowire end-fired with a dipolar aperture nanoantenna. Our nonresonant helical nanoantenna enables tunable polarization control by swirling surface plasmons on the subwavelength scale and taking advantage of the optical spin–orbit interaction. Four closely packed helical traveling-wave nanoantennas (HTNs) are demonstrated to locally convert an incoming light beam into four beams of tunable polarizations and intensities, with the ability to impart different polarization states to the output beams in a controllable way. Moreover, by near-field coupling four HTNs of opposite handedness, we demonstrate a subwavelength waveplate-like structure providing a degree of freedom in polarization control that is unachievable with ordinary polarization optics and current metamaterials.

## Nano-optics: polarization control

Gold-coated carbon nanowire spirals offer an ultra-compact means of controlling the polarization state of light. The helical structures, designed and fabricated by a French-Chinese collaboration from the FEMTO-ST Institute in France and Jinan University in China, use surface plasmons and optical spin-orbit interactions to convert an incoming linearly-polarized light beam into a circularly polarized output. Incident light is coupled into a plasmon mode of the helical nanowires via a rectangular aperture nanoantenna that perforates a thin gold layer at the base of the helix. The small size of the nanowires means that several can be packed together and the team demonstrate an array of 4 coupled structures, two with a left-handed helix and two with a right-handed helix, allowing tunable polarization control. Potential applications include use in displays, data storage, microscopy and sensing.

## Introduction

A wide variety of optical applications and techniques demand light polarization control. Traditionally, the manipulation of light polarization is realized with bulky optical elements, which utilize birefringent or dichroic materials. This field has recently experienced extraordinary advances with the emergence of plasmonics, which has offered new prospects regarding the light–matter interaction. Surface plasmon resonances in subwavelength metallic nanostructures have laid the groundwork for metamaterial research, leading to ultrathin circular polarizers^1–3^ and waveplates^4–9^ by locally tailoring the phase of light^9,10^ or generating chirality^11–18^. The nonresonant manipulation of surface plasmons has also demonstrated the ability to control light polarization^19,20^. However, both of these resonant and nonresonant structures rely on collective optical effects on arrays of nanostructures. They are therefore restricted to areas much larger than the wavelength of light, which limits design strategies and functions in polarization control. Tailoring light polarization with individual subwavelength devices would overcome these limits, but this process remains a challenge^21–23^. Silicon photonics has recently provided an on-chip waveguide-based approach to controlling the polarization state of the light scattered by an individual subwavelength structure^24,25^. However, the proposed method requires 2D devices much larger than the wavelength, which strongly limits the overall system compactness and the design degrees of freedom when considering multiple scatterers. Moreover, the individual scatterers developed so far radiate highly diverging optical waves, which may represent limitations in the implementation of subwavelength polarization optics.

A characteristic of traveling surface plasmons guided along curved trajectories is their ability to acquire orbital angular momentum (OAM)^26^ and to induce, by leakage, free-space light radiation^27^. For sharp curvatures, the OAM of the surface plasmon mode can match the spin angular momentum (SAM) of free space propagating photons, thereby opening a route towards localized beams of controlled helicity, owing to the optical spin–orbit interaction^26^. On the basis of this OAM-to-SAM transfer, we generated a helical traveling-wave nanoantenna (HTN) to produce a directional light beam of tunable polarization through a swirling plasmonic effect. Our optical nanoantenna differs from existing helical plasmonic structures^1,28–30^ by its nonresonant nature, thus extending the concept of the helical traveling-wave antenna to optics^27,31^. Our subwavelength structure enables new design degrees of freedom in polarization control. Four closely packed HTNs are shown to locally convert an incoming light beam into four beams of tunable polarizations and intensities, with the possibility of imparting different polarization states to the output beams in a controllable way. Moreover, by coupling HTNs of opposite handedness, we demonstrate a subwavelength waveplate-like structure providing a degree of freedom in polarization control that is unachievable when utilizing birefringence and dichroism in materials or artificially reproducing these properties with metamaterials.

## Results

**Fig. 1:**
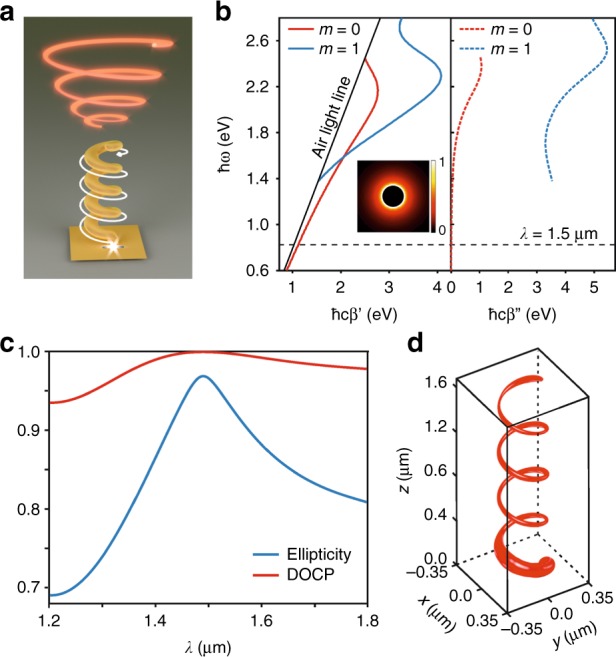
HTN as a subwavelength tunable polarizer. **a** Schematics of the HTN and its operation principle. End-fire excitation of the gold-coated helix is represented by a white spike. Under a curved trajectory along the helix, the wire mode is nonresonantly converted into a helix-guided vortex mode spreading over the cross section of the entire structure (white arrow). This mode is released in free space in the form of circularly polarized waves, owing to the spin–orbit interaction (bright red helix). **b** Dispersion relations of the *m* = 0 and *m* = 1 modes of a gold-coated carbon wire (105-nm-diameter carbon wire, 25-nm-thick gold coating). Energy is plotted versus ℏ*cβ*′ and ℏ*cβ*′′. At *λ* = 1.5 μm, only the *m* = 0 mode is guided. Figure inset: intensity plot of the *m* = 0 mode for *λ* = 1.5 μm. **c** Spectra of the ellipticity factor and DOCP of the HTN output beam. They reveal the tunable polarization properties of the nanoantenna. Depending on the wavelength, the output polarization can be tuned from elliptic to circular. **d** Amplitude of the electric current distribution along the gold-coated carbon helical wire at *λ* = 1.5 μm (numerical simulation)

Our nanoantenna consists of a narrow gold-coated carbon wire wound up in a screw-like shape forming a tiny helix (Fig. 1a). The gold-coated wire sustains a cutoff-free traveling surface plasmon, known as the TM_0_ mode^32^ (Fig. 1b). This mode, when propagated along a straight wire, features a radial polarization and thus an axially symmetrical field distribution, as shown in the inset of Fig. 1b. It is locally excited with the dipolar mode of a rectangular aperture nanoantenna that perforates a 100-nm-thick gold layer right at the helix’s pedestal. An incident wave on the back of the aperture is transmitted as a subdiffraction guided surface plasmon, which is nonradiatively converted into the wire mode of the helix. The contact between the aperture and the helix’s pedestal ensures efficient near-field coupling between the two plasmonic structures of high impedance. To identify the traveling-wave nature of the nanoantenna, we showed the intensity of the current along the metallic wire of a four-turn HTN (Fig. 1d). The thus-depicted mode closely resembles a traveling wave, as no clearly marked current nodes are evidenced. After strong attenuation along the first half-turn of the helix, the current remains almost constant over the rest of the structure, as is observed with the low-frequency traveling-wave helical antenna^31^. After half a turn, the nanoscale wire mode is nonresonantly converted into a helix-guided mode spreading over the overall structure cross section and propagating along the helix axis. This short transition length is indicative of a nanoscale plasmon coupling between the rectangle aperture nanoantenna and the helix. A far-field excitation of the helix from the aperture would require a longer distance for the helix mode to be installed, with important current inhomogeneities all along the helical wire (given the shifted position of the aperture with respect to the helix). The absence of well-defined nodes at the output end of the HTN denotes good impedance matching of the nanoantenna to vacuum (low reflection at the structure end).

In the course of propagation, the helix-guided mode acquires OAM oriented along the helix axis (0z). OAM is here transferred from the helix to the guided mode and is independent of the feed element, which enables ultracompact polarizers of subwavelength size. When circular polarization is generated by an HTN, this vortex mode (of charge 1 depending on the helix handedness) is released as freely propagating waves carrying SAM of 1 per photon (in ℏ units). The degree of circular polarization (DOCP) of the emitted waves refers to the distribution of photons prepared in the spin states +1 and −1. The DOCP is defined as $|I_{RCP} - I_{LCP}|/(I_{RCP} + I_{LCP})$, where *I*_*RCP*_ and *I*_*LCP*_ stand for the intensities of the right and left circularly polarized components of the nanoantenna radiation, respectively^4^. It corresponds to the normalized Stokes parameter *S*_3_/*S*_0_. An HTN designed to operate as a circular polarizer at *λ* = 1.5 μm has been predicted to emit light with polarization ellipticity and a DOCP peaking at 0.97 and 0.999, respectively (Fig. 1c).

We predicted from finite difference time domain (FDTD) simulations (Supplementary Section 1) that 61.2% of the light power coupled into the HTN is radiated in the far field (i.e., 38.8% of the incoupled power is absorbed by the nanoantenna due to ohmic losses). Remarkably, we found that only 14.7% of the incoupled power is dissipated by the plasmonic helix (i.e., approximately one-third of the total losses). This is in accordance with the slowly decaying current intensity of the helix’s plasmon mode installed after the first turn (Fig. 1d). The guided mode of the helix is thus weakly dissipated.

**Fig. 2:**
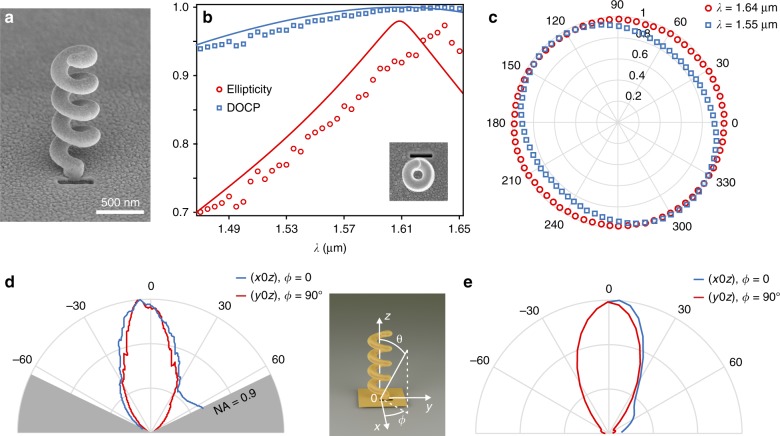
Circularly polarized beams from fabricated individual HTNs. **a** Scanning electron micrograph of an HTN. **b** Experimental spectra of the ellipticity factor and DOCP of the HTN output beam. Inset: top view of **a**. Image size: 1 μm. Solid curves: theoretical spectra (ellipticity factor and DOCP) with a 10-nm-thick gold layer covering the experimentally obtained carbon helix. **c** State-of-polarization analysis at *λ* = 1.55 μm and *λ* = 1.64 μm. The polar angle of this polarization diagram (*ϕ*) is defined in the inset of **d**. **d** Experimental radiation pattern of the HTN at the polar angle *θ*, measured in two orthogonal longitudinal planes (x0z) and (y0z) defined in the figure inset. **e** Theoretical predictions

Our fabrication of the corresponding structures started with the growth of carbon helices by focused-ion-beam-induced deposition (FIBID)^33^ on a 100-nm-thick gold film covering a glass substrate. Carbon was chosen for its excellent ability to be deposited by FIBID. The carbon helices were then coated with a thin layer of gold. The HTN was terminated by focused ion beam (FIB) milling of a rectangular aperture nanoantenna in contact with the helix pedestal and outside the winding area of the plasmonic wire (see Fig. S1 in the Supplementary Information). Figure 2a and the inset of Fig. 2b display scanning electron microscopy (SEM) images of a resulting structure. The HTNs were back-illuminated with polarized light from a tunable laser at telecommunication wavelengths, the nanoantenna output beams were measured and their polarization states were analysed (see “Methods”). The observed polarization properties (Fig. 2b, c) agree well with the theoretical model. As predicted numerically (by comparing Figs. 1c and 2b), a thinner gold coating may explain the noticeable redshift in the experimental spectra with respect to the theoretical expectations. The plasmon mode is then loaded by the high-refractive-index carbon skeleton of the helix.

The radiation pattern of the HTN was measured by imaging individual nanoantennas with a 0.9 numerical aperture microscope objective (Fig. 2d). Despite their subwavelength sizes, an HTN produces a beam centered near polar angle *θ* = 0° (see inset of Fig. 2d) with a half-width at half-maximum of 26.9° in the (x0z)-plane and 23.7° in the (y0z)-plane. (x0z) and (y0z) are defined in the inset of Fig. 2d. These experimental results agree well with theoretical predictions (Fig. 2e). They confirm the axial mode operation of the HTN^27,31^.

**Fig. 3:**
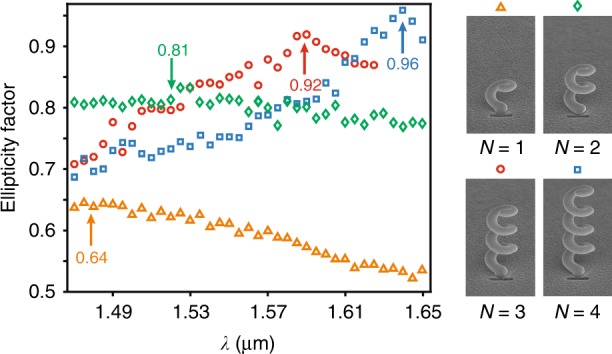
Helicity of light originates from swirling surface plasmons. Spectrum of the ellipticity factor of the HTN emission for single structures with one turn (orange triangles), two turns (green diamonds), three turns (red circles), and four turns (blue squares)

Figure 3 presents the spectrum of the far-field ellipticity factor (EF) of four helices showing an increasing number of turns. From one to four turns, the measured maximum EF varies from 0.64 to 0.96 while undergoing spectral redshift. The enhancement in the EF with the number of turns of the helix reveals the swirling plasmonic effect as the source of circular polarization. This result indicates the end-fired helix as the origin of the transmission process, which is confirmed experimentally in Supplementary Section 2.

**Fig. 4:**
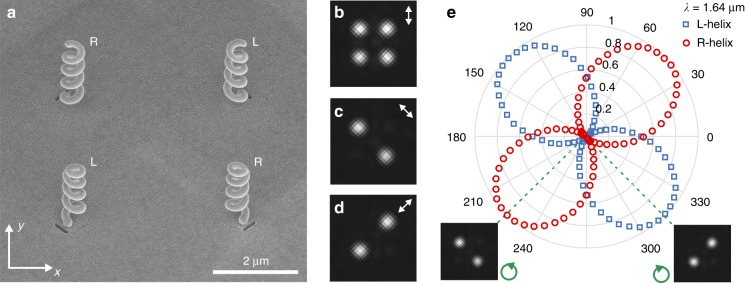
Four closely packed HTNs locally convert an incoming light beam into four beams of tunable polarizations and intensities. **a** Scanning electron micrograph of two couples of HTNs of opposite handedness and orthogonal aperture nanoantennas. The right- and left-handed HTNs are identified with the letters R and L, respectively. **b**–**d** Far-field optical images of the four-HTN device in **a** with an incoming linear polarization oriented at **b** 0°, **c** + 45°, and **d** −45° from the (0y) axis shown in **a**. **e** Helicity analysis of two HTNs of opposite handedness. The measurement is conducted by placing a rotating quarter-wave plate followed by a fixed polarizer in front of a detector and measuring the transmitted power. Insets: images of the sample with this analyser transmitting right-handed and left-handed circularly polarized light (i.e., for the quarter-wave plate to polarizer angles of ±45°)

We developed an HTN-based platform to convert linearly polarized incoming light into four closely packed circularly polarized beams: two right-handed and two left-handed circularly polarized beams (Fig. 4). To this end, we fabricated two couples of HTNs of opposite handedness, positioned at the corners of a 5-µm large fictive square, as shown in Fig. 4a. By using HTNs with various orientations of feed apertures, it is possible to tune the relative intensities of these beams by changing the polarization of the incident waves. With our HTNs of orthogonal apertures, we were able to excite all four helices (Fig. 4b; four output beams) or selectively address the right- or left-handed plasmonic structures (Fig. 4c, d, respectively; two output beams only) by rotating the input linear polarization. The polarization states of the HTN radiations were analysed by placing a rotating quarter-wave plate followed by a fixed linear polarizer in front of the camera (see Fig. S3). The resulting polarization diagram (Fig. 4e) shows that the beams produced by the right and left HTNs are right- and left-handed circularly polarized, respectively.

It is also possible to impart a different polarization state to each output beam in a controllable way simply by considering nanoantennas of various geometrical parameters (cf. Fig. S4). Moreover, as shown in Fig. 2b, the polarization state can be tuned by changing the wavelength of the incoming light. It is therefore possible to arrange at will a set of HTNs for locally converting an incoming light beam into an arbitrary distribution of directional beams of tunable polarizations and intensities, thereby obtaining unprecedented integrated devices for manipulating light polarization.

**Fig. 5:**
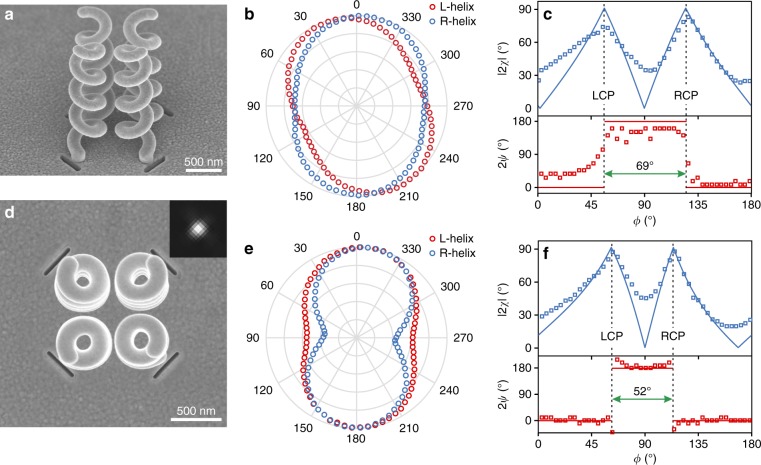
Subwavelength waveplate-like structure from four coupled HTNs. **a**, **d** Scanning electron micrographs of the subwavelength waveplate-like structure. **a** Angled view, **d** top view. **b**, **e** Polarization diagram of the nanoantenna output beam for incident polarization corresponding to *ϕ* equal to 45° and 135°, leading to the selective excitation of the two couples of HTNs of opposite handedness: **b**, *λ* = 1.61 μm, **e** *λ* = 1.47 μm. Near-field coupling between the HTNs of opposite handedness ensures parallel outcoming polarization ellipses for orthogonal incident linear polarizations. **c**, **f** Polarization angles 2*χ* and 2*ψ* of the output beam versus the polarization direction of incident linearly polarized light (Poincare sphere approach of polarization). The incident beam is impinging from the substrate at normal incidence with **c** *λ* = 1.61 μm and **f** *λ* = 1.47 μm. Owing to the oscillatory nature of light fields, the full set of incident linear polarizations is covered with a polarization angle *ϕ* ranging from 0 to 180°. Theoretical predictions are shown as solid lines (see Supplementary Section 3)

A more complex polarization response can be achieved with a spacing between the HTNs that is smaller than the wavelength, resulting in the near-field coupling of the light emission processes created by individual nanoantennas. We consider two couples of right and left HTNs with helices of opposite handedness that are spaced 560 nm apart and are made up of orthogonal apertures (Fig. 5a, d). This four-HTN structure is identical to that of Fig. 4 but with the nanoantennas packed in a volume smaller than a cubic wavelength. With this geometry, a single output beam is observed regardless of the incident polarization (inset of Fig. 5d). When the right or left HTNs are selectively excited (with two orthogonal incident linear polarizations), the output beam is no more right- or left-handed circularly polarized, as was observed in Fig. 4. By virtue of a plasmon coupling between helices of opposite handedness (Fig. S5), all four nanoantennas are excited and participate in the beam generation, regardless of the incoming polarization direction. As a result, two orthogonal linear polarizations of incident light are converted into right- and left-handed outcoming elliptical polarizations whose principal axes are parallel (Fig. 5b, e). Figure 5c, f compare the measured and calculated tilt angles 2*ψ* and ellipticity angles 2*χ* of the outcoming polarization (Poincare sphere approach) as a function of the direction angle *ϕ* of the incident linear polarization at two different wavelengths (1.61 μm and 1.47 μm), respectively. The measured curves in Fig. 5c, f reveal the theoretically anticipated tuning of the angular spacing Δ*ϕ* between the two right- and left-handed outcoming circular polarizations. Whereas Δ*ϕ* is fixed at 90° with conventional quarter-wave plates, it is here equal to 69° at *λ* = 1.61 μm and decreases to 52° at *λ* = 1.47 μm. This tunability in polarization manipulation is not standard at all. It provides a degree of freedom in polarization control that is unachievable when utilizing or artificially reproducing birefringent and dichroic materials. It arises from the possibility of generating circular polarizations from the combination of elliptically polarized waves of opposite handedness, parallel principal axes and tunable intensities (see Supplementary Section 3). By spectrally detuning the HTNs, the outcoming polarization ellipticities are modified, thereby resulting in a controlled and tunable angular spacing Δ*ϕ*. The above-described polarization properties appear to be robust to fabrication defects.

## Discussion

We demonstrated that circularly polarized light can be locally achieved via an individual plasmonic nanoantenna designed by transposing the concept of a helical traveling-wave antenna to optics. The HTN is a subwavelength structure capable of producing a background-free directional light beam of tunable polarization and intensity, which is unprecedented to the best of our knowledge. Relying on the spin–orbit interaction of light, HTNs lead to ultracompact, versatile and robust polarization optics, enabling new degrees of freedom in light polarization control. First, HTNs arranged at will on a surface have been demonstrated to locally convert an incoming light beam into an arbitrary distribution of directional beams of tunable polarizations and intensities. Different polarization states are here imparted to the output beams in a controllable and tunable way. This represents an unprecedented integrated function in light polarization control. Second, by near-field coupling four HTNs of opposite handedness, we obtain a subwavelength waveplate-like structure providing a degree of freedom in polarization control that is unachievable with ordinary polarization optics and current metamaterials (i.e., devices utilizing or artificially reproducing birefringence and dichroism). By scaling down the nanoantenna dimensions, this polarization control could be extended to shorter wavelengths at the expense of weaker plasmonic effects and higher absorption in metals. Silver or aluminum would thus be preferred in the visible portion of the spectrum. Taken as individual or coupled nanostructures, HTNs may pave the way towards highly integrated polarization-encoded optics, particularly for the generation and control of spin-encoded photon qubits in quantum information and opto-spintronics. More generally, our results lay a solid basis for subwavelength polarization optics, thus opening new perspectives in photonic information processing, polarimetry, miniaturized displays, optomagnetic data storage, microscopy, sensing and communications, etc.

## Methods

### Simulations

All numerical simulations of the HTN emission process are realized using the 3D FDTD method. The plasmonic helix geometry considered in this study consists of a 105-nm-diameter carbon wire wound up in the form of a four-turn corkscrew-type structure and covered with a 25-nm-thick gold layer. The resulting helix has a 505-nm outer diameter and is 1.66 μm high. It is positioned on a pedestal with a 105-nm-diameter and 100-nm-high carbon rod, the cylindrical lateral side of which is covered with a 25-nm-thick gold layer. The helix pitch angle is ~20.7°. The helix pedestal lies on a 100-nm-thick gold layer deposited onto a glass substrate. The rectangular aperture nanoantenna, with a width and length equal to 40 nm and 370 nm, respectively, is engraved in the metal layer. Its center is located at *x* = *y* = 0. *z* = 0 corresponds to the upper surface of the gold layer that covers the glass substrate. To excite the HTN, a Gaussian beam (beam waist equal to 1.5 μm) impinges onto the rectangular aperture nanoantenna at normal incidence from the backside.

The spectral response of the HTN is obtained with a Gaussian excitation described by a single temporal pulse. The time-varying electric field is calculated at a single cell located on the helix axis, 4 μm away from the end of the helix, along (0z). The spectra of the vector components *E*_*x*_ and *E*_*y*_ are calculated by Fourier transforming this result. From these results, the EF is deduced as a function of the wavelength. The model used for the spectrum calculations consists of a volume spanning 4.55 μm in the *x* and *y* directions perpendicular to the longitudinal helix axis. It extends 2 μm below the gold layer in the glass substrate and terminates 4.3 μm beyond the top of the helix in air. All six boundaries of the computation volume are terminated with perfectly matched layers to avoid spurious unphysical reflections around the structure. The nonuniform grid resolution varies from 30 nm for portions at the periphery of the simulation to 5 nm within and near the helix and the aperture nanoantenna.

In all the calculations conducted in the continuous wave regime, the wavelength is 1.5 μm. The distribution of the current amplitude within the helix is plotted by integrating the simulated optical current density across the gold coating of the helix-shaped carbon wire for each curvilinear coordinate along the wire. The HTN geometry and mesh grid parameters remain unchanged. The computation volume spans 2.1 μm in the *x* and *y* directions. It extends 0.75 μm below the gold layer in the glass substrate and terminates 2.61 μm beyond the top of the helix in air.

### Fabrication

The HTNs were fabricated in three steps using FIBID technology and FIB milling (Dual Beam SEM/FIB FEI Helios 600i). A helical carbon skeleton was fabricated by FIBID onto a commercial 100-nm-thick gold film. For operation at 1.63 μm (experimentally), the geometrical parameters are a pitch angle of ~19.8°, a radius of 155 nm and a pitch length of 350 nm. The structure was covered with a thin smooth layer of gold sputter-deposited by glancing angle deposition. Then, a 370-nm-long and 40-nm-wide rectangular aperture nanoantenna was milled in contact with the helix pedestal (see Fig. S1).

### Characterization

A schematic diagram of the experimental setup is presented in Fig. S3. It is mounted onto a Nikon 239 TE2000 inverted microscope. Light of tunable wavelength, ranging from 1.47 to 1.65 μm, emerges from a tunable laser source (Yanista Tunics-T100S) and is coupled to a single-mode polarization-maintaining fiber (P3-1500PM-FC-2, Thorlabs). It is collimated by an achromatic reflective fibre collimator (RC08APC-P01, Thorlabs) and focused onto the plasmonic structures with either a (25×, 0.4) microscope objective for the study of individual HTNs or a (4×, 0.1) objective for the study of four-coupled HTN structures.

The rectangular aperture nanoantenna is illuminated from the backside, and light radiation occurs from the plasmonic helix. To measure the polarization diagram of the nanoantenna, the polarization of the incident collimated wave is manipulated using a fixed polarizer (LPNIR100-MP2) and a half-wave plate (AHWP05M-1600, Thorlabs) positioned between the collimator and the objective. The half-wave plate is mounted onto a motorized stage (PRM1Z8, Thorlabs) to be accurately rotated with respect to the polarizer. The plasmonic structures are imaged with a (50×, 0.65) infrared objective from Olympus (LCPlanN) coupled to an infrared camera (GoldEye model G-033, Allied Vision Technologies GmbH) and a proper field lens. To analyse the polarization state of the light emitted by our plasmonic nanoantennas, either a rotating linear polarizer or a fixed polarizer coupled to a rotating quarter-wave plate (see inset) are inserted in front of the camera (linear polarizer: LPNIR100-MP from Thorlabs; quarter-wave plate: AQWP05 M-1600 from Thorlabs; and motorized stage: UE16CC from Newport). The rotating linear polarizer is used to measure the polarization diagram of the output beam, from which we deduce an EF. The corresponding DOCP value can be derived from the EF according to the equation DOCP = 2EF/(1+EF^2^) demonstrated in Supplementary Section 4. In Fig. 2, the spectra are obtained by analysing the state of polarization at each wavelength. The incident polarization is oriented perpendicularly to the long axis of the rectangular aperture nanoantenna to excite its fundamental plasmon mode. Right- and left-handed circular analysers are obtained by orienting the fast axis of the quarter-wave plate at +45° and −45° relative to the polarizer axis, respectively. For emission pattern measurements (Fig. 2), a lens is inserted behind the microscope objective to Fourier transform the image of the nanoantenna: an image of the back focal plane of the objective is projected onto the camera. A (60×, 0.9) microscope objective is then used to image the HTN.

## Supplementary information

Supplementary Information-Subwavelength polarization optics via individual and coupled helical travelling-wave nanoantennas.
