# Supplementary material for: Subwavelength polarization optics via individual and coupled helical traveling-wave nanoantennas
Source: Light Sci Appl. 2019 Aug 28;8:76. doi: 10.1038/s41377-019-0186-2 (PMC6804798; doi:10.1038/s41377-019-0186-2)
Supplement: Supplementary file 1 — Supplementary Information-Subwavelength polarization optics via individual and coupled helical travelling-wave nanoantennas. [file 41377_2019_186_MOESM1_ESM.pdf]

Supplementary Information

Subwavelength polarization optics via individual and coupled helical travelling-wave nanoantennas

Mengjia Wang,[1] Roland Salut,[1] Huihui Lu,[2**] Miguel-Angel Suarez,[1]

Nicolas Martin,[1] and Thierry Grosjean,[1*]

[1] *FEMTO-ST Institute UMR 6174, Univ. Bourgogne Franche-Comté CNRS - Besancon, France*

[2] *Guangdong Provincial Key Laboratory of Optical Fiber Sensing and Communications, Department of Optoelectronic Engineering, Jinan University, Guangzhou 510632, China*

[3] **Email: thierry.grosjean@univ-fcomte.fr; **email: thuihuilu@jnu.edu.cn*

1 Numerical estimation of the optical losses of the HTN.

In order to evaluate the losses associated with a single HTN, we calculated by FDTD simulation the total power funnelled into the rectangular nanoaperture (P_{inc}) and that radiated by the HTN into the free space (P_{far}). Their ratio $\eta = P_{far}/P_{inc}$ can be deduced from Poynting vector flow calculations:

$$\eta = \frac{\int\int \Re\left(\mathbf{E}_{far} \times \mathbf{H}^{*}_{far}\right) R_0^2 \sin\theta d\theta d\phi}{\int\int \Re\left(\mathbf{E}_{inc} \times \mathbf{H}^{*}_{inc}\right) dxdy}, \tag{S1}$$

where ($\mathbf{E}_{far}, \mathbf{H}_{far}$) and ($\mathbf{E}_{inc}, \mathbf{H}_{inc}$) define the electromagnetic optical fields far way from the structure (at R_0=150 μm) and in a plane 5 nm beyond the entrance (glass/metal) interface of the metal layer, respectively. (θ, ϕ) are spherical angles. The losses can then be quantified by $1 - \eta$.

2 End-fire excitation of the plasmonic helix

Fig. S2 shows that polarization properties of the helical travelling-wave nanoantenna (HTN) rely on the end-fire excitation of its plasmonic helical wire. We studied the ellipticity factor of the output beam for three nanoantenna configurations involving three different excitation schemes from a rectangular aperture nanoantenna. The ellipticity factor peaking at a value larger than 0.92 when the aperture nanoantenna is in contact to the helix pedestal drops down to 0.73 when it is removed 185 nm away from the helix. When the rectangular nano-aperture antenna is rotated by 90°, i.e., when the orientation of its dipolar plasmon mode becomes orthogonal to the radially-polarized wire mode of the plasmonic helix, the ellipticity factor of the HTN output beam is reduced to 0.32. The 3D vectorial near-fields produced by the rectangular aperture nanoantenna may explain, in the latter case, the non-null coupling between the orthogonal nano-aperture and the helix.

3 Analytical model of our subwavelength waveplate-like structure (four coupled HTNs)

The polarization properties of our subwavelength waveplate-like structure (Fig. 5) relies on the combination of two elliptically polarized waves of opposite handedness, and the control of their respective intensities. Such a configuration can be simply modeled by the interference of two co-propagating plane waves described by parallel polarization ellipses.

Owing to the field projection rules defined by the two pairs of orthogonal aperture nanoan-

tennas, the electric fields $\mathbf{E}_1$ and $\mathbf{E}_2$ of these two waves can take the following form:

$$\mathbf{E}_1 = \sin(\phi - \frac{\pi}{4})\,(1, ib_1, 0)\exp\left[-i(\omega t - wz)\right], \tag{S2}$$

$$\mathbf{E}_2 = \cos(\phi - \frac{\pi}{4})\,(1, -ib_2, 0)\exp\left[-i(\omega t - wz)\right]. \tag{S3}$$

The two light waves show the same wave vector $(0, 0, w)$. (x, y, z) are the space coordinates, ω is the angular frequency, and t refers to time. b_1 and b_2 are two positive constants smaller than 1, called ellipticity factors.

Circular polarization arises when:

$$\mathbf{E}_1 + \mathbf{E}_2 = K\,(\pm 1, i, 0)\exp\left[-i(\omega t - wz)\right], \tag{S4}$$

where K is a constant, thus imposing:

$$\tan(\phi) = \left|\frac{1 \pm b_2}{1 \mp b_1}\right| + \frac{\pi}{4}, \tag{S5}$$

Right and left circular polarizations are then obtained for two specific values of ϕ that are dependent on the ellipticity factors b_1 and b_2 of the two initial waves.

More generally the electric field resulting from the wave combination can be written

$$(E_x, E_y, 0) = \sin(\phi - \frac{\pi}{4})(1, ib_1, 0) + \cos(\phi - \frac{\pi}{4})(1, -ib_2, 0). \tag{S6}$$

We can consider this total field as an elliptically polarized wave whose major and minor radii take respectively the form :

$$a = \frac{\sqrt{2}}{2}\left[|E_x|^2 + |E_y|^2 + \sqrt{|E_x|^4 + |E_y|^4 + 2|E_x|^2|E_y|^2\cos(2\Delta\xi)}\right]^{\frac{1}{2}}, \tag{S7}$$

$$b = \frac{\sqrt{2}}{2}\left[|E_x|^2 + |E_y|^2 - \sqrt{|E_x|^4 + |E_y|^4 + 2|E_x|^2|E_y|^2\cos(2\Delta\xi)}\right]^{\frac{1}{2}}, \tag{S8}$$

where $\Delta\xi$ refers to the phase difference between E_x and E_y [3]. From Eqs. S7 and S8, we anticipated the polarization state of the four-HTN radiation, as a function of the projection angle ϕ (Fig. S6). We considered the particular case where $b_1 = b_2$, i.e., two waves of identical ellipticities and opposite handedness. When these two waves are circularly polarized, the angular spacing $\Delta\phi$ between right and left polarizations is 90°. The resulting polarization manipulation is similar to that of a rotating polarizer in front of quarter-wave plate. In that case, ϕ refers to the projection angle of an incident linearly polarized wave onto the crystalline axes of the retardation plate.

Polarization control deviates from this well-known conventional configuration when we consider two elliptically polarized waves (b_1 and b_2 become smaller than 1). Depending on the ellipticity factor of the two initial waves, the angular spacing $\Delta\phi$ between right and left circular polarizations decreases down to 53° when $b_1 = b_2 = 0.5$ and 11.5° when $b_1 = b_2 = 0.1$. The four-HTN structure would thus lead to a near-switching effect of circular polarization handedness while rotating linear polarization of an incoming light. By implementing the polarization states shown in Figs. 5b and 5e in our model, we found the polarization manipulations described by the solid lines of Figs. 5c and 5f, respectively.

4 Relation between the ellipticity factor and degree of circular polarization

Supposing an arbitrary elliptic polarization state, we have the following electric field vector:

$$\mathbf{E} = C\,(1, \pm iEF, 0)\;\;(i = \sqrt{-1}) \tag{S9}$$

in proper Cartesian coordinate frames, where EF stands for the ellipticity factor and C is a constant.

It can be decomposed as the linear combination of left and right circularly polarized fields:

$$\mathbf{E} = \alpha_{LCP}\,(1, +i, 0) + \alpha_{RCP}\,(1, -i, 0)\,, \tag{S10}$$

where α_{LCP} and α_{RCP} are the linear combination coefficients associated with the left and right circular polarizations, respectively. We thus have:

$$\alpha_{LCP} + \alpha_{RCP} \;=\; C \tag{S11}$$

$$\alpha_{LCP} - \alpha_{RCP} \;=\; \pm EF \cdot C \tag{S12}$$

The corresponding degree of circular polarization ($DOCP$) can be calculated as follows:

$$DOCP = \frac{|I_{RCP} - I_{LCP}|}{I_{RCP} + I_{LCP}} = \frac{||\alpha_{RCP}|^2 - |\alpha_{LCP}|^2|}{|\alpha_{RCP}|^2 + |\alpha_{LCP}|^2}. \tag{S13}$$

Substituting Eqs. S11 and S12 into Eq. S13 and reforming, we obtain:

$$DOCP = \frac{EF}{1 + EF^2}, \tag{S14}$$

which had been used to derive the $DOCP$ value from the measured polarization diagram.

Supplementary References

1. M. Esposito, *et al.*, *Nano Lett.* **16**, 5823 (2016).

2. J. D. Kraus, R. J. Marhefka, A. S. Khan, *Antennas and wave propagation* (Tata McGraw-Hill Education, 2006).

3. C. Balanis, *Antenna theory: analysis and design* (John Wiley & Sons, New-York, 1997).

4. M. Esposito, *et al.*, *Nano Lett.* **16**, 5823 (2016).

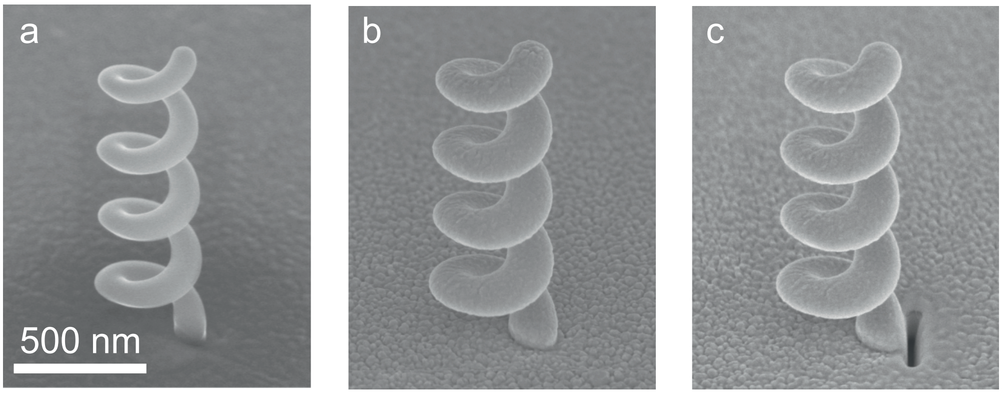

Figure S1: **Fabrication of the helical travelling-wave nanoantenna (HTN): three steps.** Scanning electron micrographs of the subwavelength structure after **a** fabrication of the carbon helix skeleton by FIBID [4], **b** gold deposition onto the helix skeleton, and **c** fabrication of rectangular nano-aperture antenna by FIB milling.

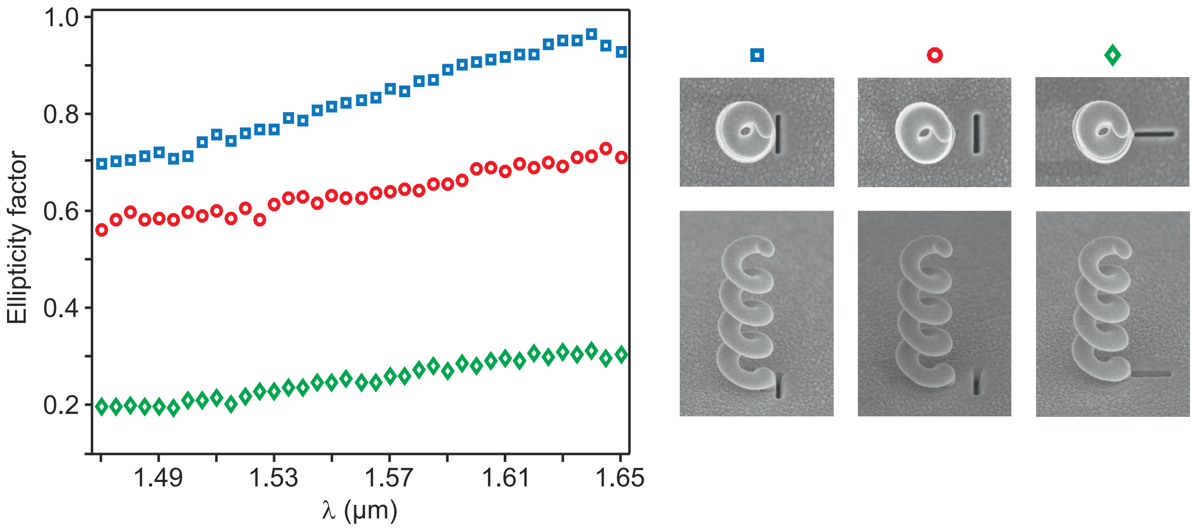

Figure S2: **Circularly polarized light originates from an end-fired plasmonic helix**. Spectrum of the polarization ellipticity factor of the HTN output beam for a rectangular nano-aperture antenna in contact to the helix pedestal (blue squares), 185 nm away from the helix pedestal (red circles), and turned by 90° regarding the two first cases (green diamonds).

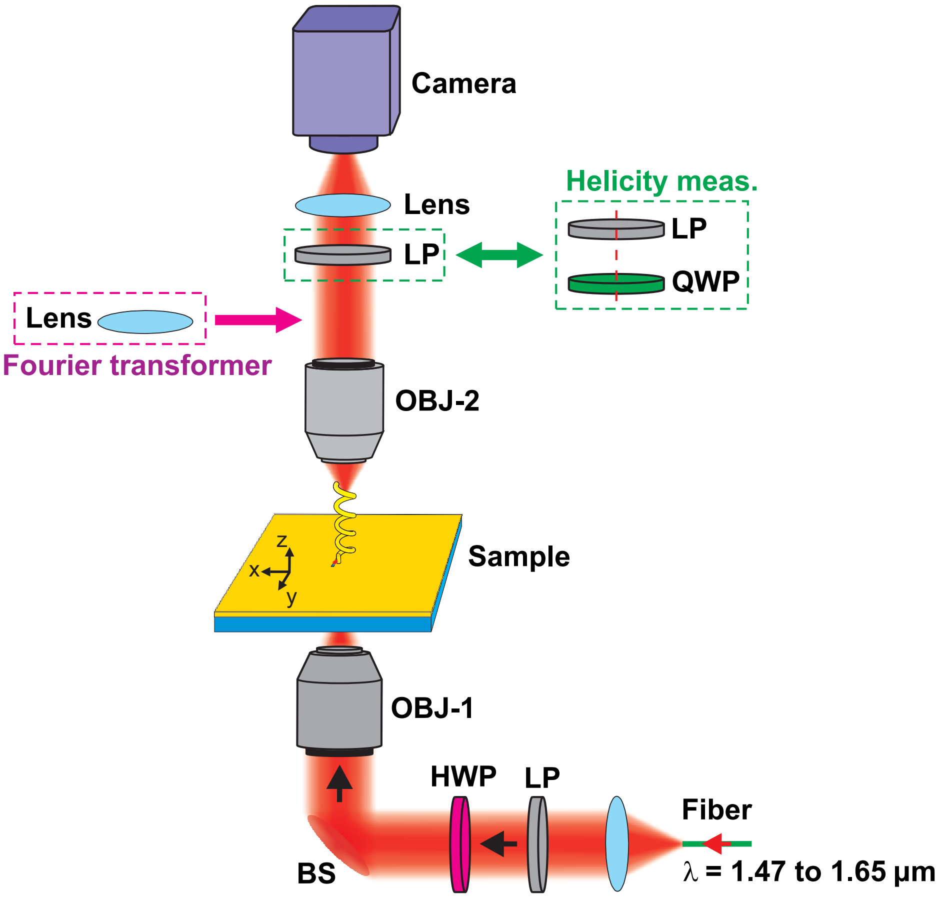

Figure S3: **Experimental setup for optical characterization**. Measurement of the polarization properties of the HTN. The beam splitter does not affect the polarization state of the reflected light. BS: Beam Splitter, LP: Linear Polarizer, HWP: Half-Wave Plate, OBJ: Objective, QWP: Quarter-wave plate

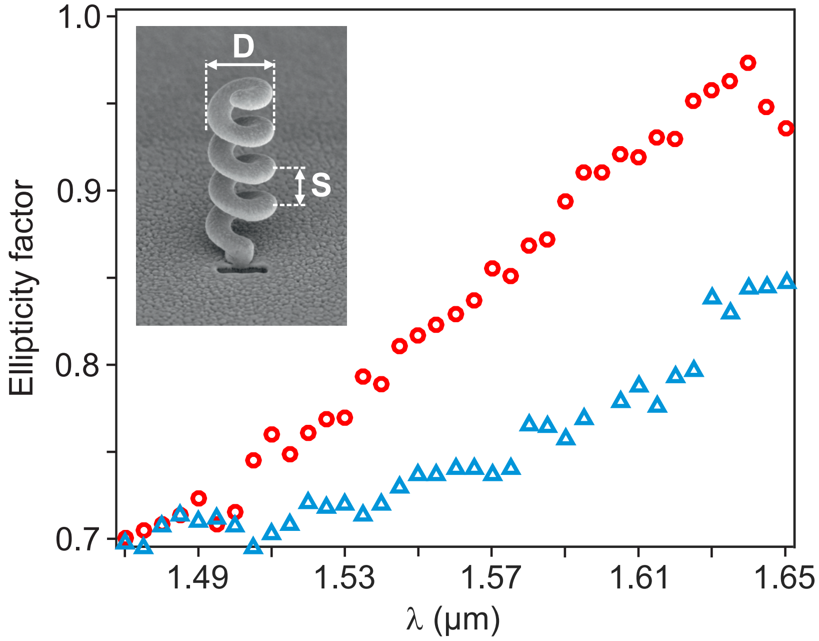

Figure S4: **Tuning the polarization properties of a HTN by modifying its geometrical parameters**. Spectrum of the polarization ellipticity factor of two HTNs showing different geometrical parameters: D=523 nm, S=360 nm (red circles) and D=553 nm, S=380 nm (blue triangles). The wire diameter remains unchanged. D and S are defined in the figure inset.

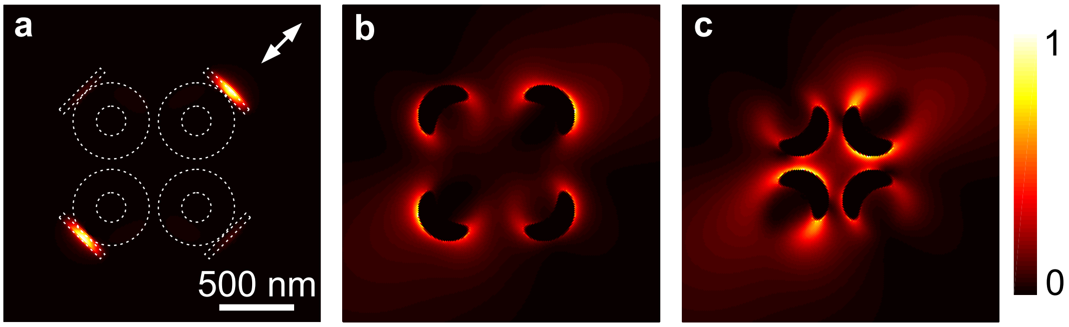

Figure S5: **Coupled HTNs**. Simulated intensity distribution of the electric optical field in three transverse planes that are positioned **a** 10 nm, **b** at two turns and **c** at two and half turns beneath the ground plane of the nanoantennas. The polarization of the incoming light is shown by the white arrow in **a**. The apertures and the helices are also outlined in white in **a**. In that configuration, only the right-handed HTNs are selectively addressed by the incoming wave (**a**). By virtue of a plasmon coupling between the HTNs of opposite handedness, all the helices are excited and lead to the generation of a right-handed elliptically polarized output beam (and no-more a right-handed circularly polarized beam, as for the individual right-handed helices). A mirror symmetric configuration is obtained when the left-handed HTNs are selectively addressed by the linearly polarized incoming light (by turning the polarization direction by 90°). Two outcoming elliptical polarizations of opposite handedness and parallel axes are thus obtained for two orthogonal incident linear polarizations.

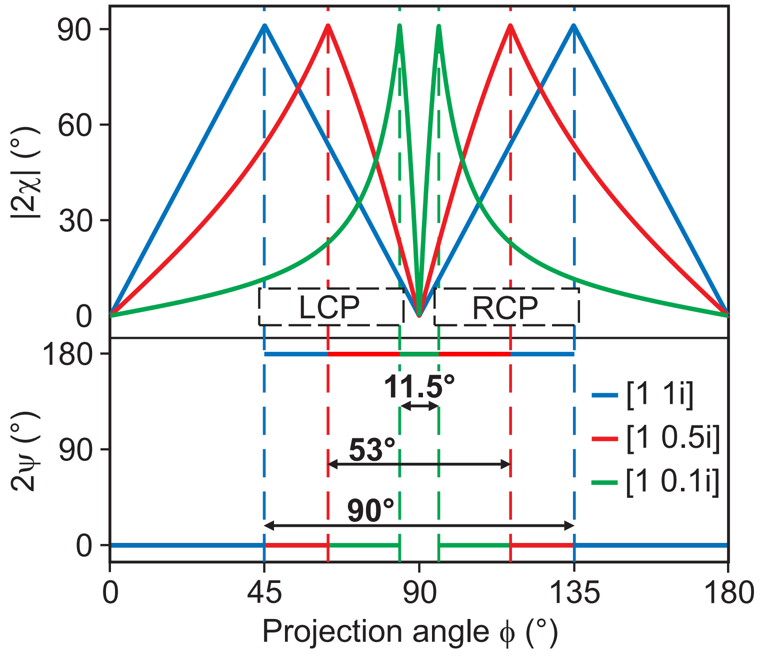

Figure S6: **Theoretical anticipation of our subwavelength waveplate-like structure**. Prediction of the polarization state of the output beam (i.e., the polarization angle $|2\chi|$ and 2ψ on the Poincare sphere), as a function of the projection angle ϕ defining the HTN emission intensities. Three ellipticity factors are considered: $b_1 = b_2 = 1$ (blue curves), $b_1 = b_2 = 0.5$ (red curves) and $b_1 = b_2 = 0.1$ (green curves).
